# The Interplay between the Gut Microbiome and the Immune System in the Context of Infectious Diseases throughout Life and the Role of Nutrition in Optimizing Treatment Strategies

**DOI:** 10.3390/nu13030886

**Published:** 2021-03-09

**Authors:** Selma P. Wiertsema, Jeroen van Bergenhenegouwen, Johan Garssen, Leon M. J. Knippels

**Affiliations:** 1 Danone Nutricia Research, Uppsalalaan 12, 3584 CT Utrecht, The Netherlands; jeroen.vanbergen@danone.com (J.v.B.); johan.garssen@danone.com (J.G.); leon.knippels@danone.com (L.M.J.K.); 2 Division of Pharmacology, Utrecht Institute for Pharmaceutical Sciences, Faculty of Science, Utrecht University, 3584 CT Utrecht, The Netherlands

**Keywords:** infectious diseases, microbiome, gut health, mucosal immunity, systemic immunity, nutrition

## Abstract

Infectious diseases and infections remain a leading cause of death in low-income countries and a major risk to vulnerable groups, such as infants and the elderly. The immune system plays a crucial role in the susceptibility, persistence, and clearance of these infections. With 70–80% of immune cells being present in the gut, there is an intricate interplay between the intestinal microbiota, the intestinal epithelial layer, and the local mucosal immune system. In addition to the local mucosal immune responses in the gut, it is increasingly recognized that the gut microbiome also affects systemic immunity. Clinicians are more and more using the increased knowledge about these complex interactions between the immune system, the gut microbiome, and human pathogens. The now well-recognized impact of nutrition on the composition of the gut microbiota and the immune system elucidates the role nutrition can play in improving health. This review describes the mechanisms involved in maintaining the intricate balance between the microbiota, gut health, the local immune response, and systemic immunity, linking this to infectious diseases throughout life, and highlights the impact of nutrition in infectious disease prevention and treatment.

## 1. Infections

In the Western world, the incidence of infectious diseases has reduced significantly during the past decades owing to improved hygiene, vaccination, and the use of antibiotics [1]. In the developing world, however, nearly one-third of deaths are still associated with infectious diseases. In addition, infections still pose a significant risk for vulnerable people, such as infants and the elderly. Upper respiratory tract infections are the most common disease for which individuals seek medical care and, in elderly people, both influenza and pneumonia are still a common cause of death [1]. The World Health Organization states that infectious enteric disease is one of the main causes of death and, according to the Global Burden of Diseases, Injuries, and Risk Factors Study of 2015, infectious diarrhea is a major cause of death worldwide, with a large percentage of these deaths occurring in the under 5 age group [2,3].

## 2. Protection from Infections in the Gastro-Intestinal Tract: The Gut Microbiota and Epithelial Barrier

There are three main hurdles that pathogens need to overcome to cause an infection in the gastro-intestinal (GI) tract: the intestinal microbiota, the intestinal epithelial layer, and the mucosal immune system [4].

The gut microbiota consists of a multispecies microbial community, consisting of bacteria, fungi, and viruses, living within a particular niche in synergy with the host [5]. Gut microbes and mammals have coevolved, so while microbes get a habitat to florish, the microbes regulate various host physiological functions, including regulating protective immunity against pathogens [6]. The composition of the gut microbiota is influenced by many factors, such as genetics; gender; age; socio-economic factors; nutrition; stress; and environmental factors, such as pollutants, antibiotics, and others—the so-called exposome [7]. Factors that disturb the microbial community structure and function, like the use of antibiotics, give space to opportunistic pathogens to colonize, grow, and persist [8]. There are several mechanisms in which the microbiota ensures the prevention of colonization, overgrowth, pathogen-induced damage, and subsequent infection of the host. One mechanism is referred to as colonization resistance, where the commensal microbiota and invading microorganisms compete for resource availability, or niche opportunity, either in terms of nutritional or functional space [8,9,10]. To allow for this competition, bacterial cells continuously sense the environment using signaling molecules accumulated during bacterial replication, in that way monitoring population density and adjusting their gene expression accordingly, a mechanism called quorum sensing [5,11]. The chemical signals lead to phenotypic changes of the bacteria that are associated with adherence, motility, and intestinal density, or with the excretion of protective compounds. The quorum sensing mechanism is used by commensals to ensure gut homeostasis, but is also used by pathogens to minimize host immune responses and increase pathogenicity [4]. Alterations to microbiota community structure, or a non-beneficial microbiota composition, potentially induced by diet, stress, and antibiotic and drug treatment, change the overall dynamics between the microbiota and host to result in low-grade inflammation, reduced colonization resistance, and altered infection susceptibility [12].

Next to the gut microbiota, the gut epithelial barrier plays a crucial role in protecting the host from infections by pathogens [13]. This physical barrier that separates the commensal bacteria in the gut from the underlying tissues is a monolayer of cells joined through tight-junction-protein complexes. The assembly of tight-junction complexes is a dynamic process, which can be disrupted by certain bacteria through the release of toxins [8]. In addition, the epithelial cell layer is reinforced by a layer of mucus. This mucus lining of the epithelial barrier is among the first defense mechanisms of the intestinal epithelia against bacterial invasion, by preventing luminal and mucosal microbes from directly interacting with epithelial cells [4]. In addition to its function as a biophysical barrier, mucus also acts as a reservoir for host-produced antimicrobial molecules such as secretory IgA and defensins [14]. Mucus production and degradation are governed via an intricate interaction between host and microbes regulated through host recognition of microbe-associated molecular patterns (MAMPs) and bacterial metabolites, and are thus susceptible to changes in the composition of the indigenous microbiota [15]. Between mucus and microbes exists a reciprocal relationship where both changes in the host inflammatory state and changes in the microbiota composition can contribute to alterations in mucus production and composition, leading to increased susceptibility to infection [16].

The continuous interaction between the gut microbiota and intestinal epithelium leads to constant immune signaling [17]. Regulation of this immune response, together with epithelial barrier integrity and permeability in the presence of commensal bacteria and invading pathogens, is essential for the maintenance of intestinal homeostasis. If this process is impaired, it can result in inflammation and infection.

## 3. Protection from Infections: Interaction between the Gut Microbiota and the Local Immune System

The immune response plays a crucial role in the susceptibility to and persistence and clearance of infections. The immune system is comprised of two parts: the innate immune system and the adaptive immune system [18]. The innate immune system provides nonspecific protection by several defense mechanisms, which include physical barriers such as the skin and mucous membranes; chemical barriers like enzymes and antimicrobial proteins; and innate immune cells including granulocytes, macrophages, and natural killer cells [19]. The cells of the adaptive immune system, T- and B-lymphocytes, recognize and respond to specific foreign antigens. T cells recognize infectious agents that have entered into host cells. This type of adaptive immunity depends on the direct involvement of cells and is, therefore, referred to as cellular immunity. In addition, T cells play an important role in regulating the function of B cells that secrete antibodies and proteins that recognize specific antigens. Because antibodies circulate through the humours (i.e., body fluids), the protection induced by B cells is termed humoral immunity [20].

Development of the immune system and efficacy of the immune response go hand in hand with development and composition of the gut microbiota. The evidence for this comes from comparing age- and sex-matched germ-free raised mice that have no commensal microflora, with conventionally raised animals of the same strain and with germ-free mice raised with a defined microbiota, so-called gnotobiotic mice. In particular, the use of gnotobiotic mice has elevated our understanding of the effect of single bacterial strains, consortium of strains, specific microbe-expressed genes, and microbial-produced metabolites on intestinal homeostasis and local and systemic immunity [21]. Insights from these studies highlight that innate immunity plays a key role in the first recognition of and response to microbiota-derived products. Innate immunity in the gut begins with the single layer of intestinal epithelial cells (IECs) that are directly exposed to luminal contents and microbial products. The crucial equilibrium between host and microbes is safe guarded through the recognition of microorganisms via pattern-recognition receptors (PRRs). PRRs constitute a large family of extracellular and intracellular receptors that recognize specific microbe-associated molecular patterns (MAMPs). PRRs include TLRs, C-type lectin receptors (CLRs), nucleotide binding oligomerization domain (NOD)-like receptors (NLRs), and cytosolic sensors of DNA and RNA. Activation of PPRs leads to induction of chemokines and cytokines necessary to orchestrate a protective immune response [22]. MyD88 is an important adaptor molecule downstream of PRR signaling, linking PRR activation with activation of the transcription factor NF-ĸB, which is a master regulator of inflammation. Deficiency in MyD88 thus leads to a compromised immune response and an increased susceptibility to infections [23,24,25]. However, inappropriate activation of PRRs may lead to overzealous immune responses and even to inflammatory disease and autoimmunity; therefore, PRR responses are tightly regulated via positive and negative feedback loops and cross-regulation, which have been described in a previous review [26]. In addition, IECs secrete anti-microbial peptides (AMPs), which are innate immune effector molecules with bactericidal, anti-inflammatory, and anti-endotoxic properties [27]. AMPs are essential components of the innate immune defense and work to limit pathogen interaction with the epithelium. Their expression can be down-regulated by certain pathogens and enhanced by the presence of specific microorganisms and, therefore, the composition of the microbiota is key in shaping the innate immune response [4,8].

Another mechanism through which the microbiota steers the immune response is through the formation of metabolites produced by the gut microbiome from dietary components, host products, or other microbial metabolites [28]. A broad assortment of microbial metabolites mediate many of the protective functions of commensal bacteria. Metabolites such as short-chain fatty acids (SCFA), tryptophan metabolites, and bile acid derivatives have all been shown to possess immunoprotective abilities. SCFAs enhance the production of antimicrobial peptides and mucus by specialized intestinal epithelial cells and stimulate the maturation and expansion of colonic regulatory T cells, which dampen local inflammatory responses to the microbiota [29]. SCFAs support intestinal homeostasis in the colon by modulating the epithelial barrier and support repair of intestinal cells through the induction of proliferation and differentiation of these cells [5]. In addition SCFAs are important in the proliferation of innate lymphoid cells (ILC3), which release IL22, important in the induction of antimicrobial molecules by epithelial cells [30].

Tryptophan metabolites and especially indoles are derived from the commensal fermentation of dietary tryptophan and function as ligands for the aryl hydrocarbon receptor (AhR), a receptor important in the maintenance of intestinal homeostasis, and loss of these metabolites is associated with the occurrence of inflammatory bowel disease [31,32].

Bile acid derivatives support intestinal homeostasis and impose effects on a plethora of host functions through their activation of farnesoid X receptor (FXR) and G protein-coupled bile acid receptor (TGR5) [33]. Bile acid derivates are metabolically derived from bile acids through the action of bacterial bile salt hydrolases (BSHs), which are expressed by specific bacteria phyla, and loss of the abundance of BSH genes is associated with the occurrence of inflammatory bowel disease [34].

The above indicates that mucosal homeostasis in the gut is a delicate balance between gut microbiota, microbial metabolites, and host factors. This continuous interaction leads to a tightly regulated physiological low-grade inflammatory status, maintaining optimal host defense, which affects the susceptibility to infections [35,36].

## 4. Protection from Infections: The Effect of the Gut Microbiota on Systemic Immunity

It is increasingly recognized that the gut microbiome, besides regulating the local mucosal immune system, also affects innate and adaptive cell-mediated systemic immune responses through a variety of mechanisms [37]. One mechanism involves the release of microbial soluble products, which translocate into the circulation and influence the activation of immune cells in the periphery [29]. Indeed, resident immune cells in organs distal to the gut can directly sense circulating microbial derived factors, and the absence of microbiota-derived signaling molecules causes alterations in immune function that lead to susceptibility to systemic infection [5].

At this time, perhaps the best characterized mechanism by which the gut microbiome influences the systemic immune response is its influence on the T cell compartment of the adaptive immune system [38]. It has been shown that gastrointestinal tract microbiota can affect the differentiation of T cell populations into T-helper (Th) Th1, Th2, and Th17 cells or into T cells with a regulatory phenotype [39,40]. Specifically, butyrate as a SCFA promotes this differentiation of peripherally induced regulatory T cells and, in this manner, is capable of inhibiting the development of systemic inflammation [41]. SCFAs are also capable of reprogramming the metabolic activity of cells, leading to the induction of regulatory B cells and inhibiting the generation of Th17 cells by pentanoate, which may be relevant in both inflammatory bowel diseases and autoimmune diseases [42]. In addition, microbiota-derived ATP can induce the expansion of Th17 cells, tryptophan breakdown products can lead to an increase of intraepithelial CD4 + CD8αα + T cells, and bacterially derived polysaccharides can prime regulatory T cells [43]. Through its ability to induce regulatory populations, the microbiome can support the suppression of inflammatory responses [44].

Studying host–pathogen interactions, it was shown that commensal activation of memory T cells and their trafficking to inflamed sites is necessary to protect from infection with bacterial pathogens [45]. Moreover, active control of IL10-mediated anti-inflammatory responses by commensals is important to protect from an infectious insult. This effect could be reproduced using specific toll-like receptor (TLR) agonists, which reduced IL10 production, rendering mice more resistant to infection via increased bacterial clearance and enabling proper inflammatory responses [46].

The ability of signaling molecules released by the microbiota to enter the circulation also allows resident bacteria in the gut to already modulate the immune system during immune cell development during hematopoiesis and, in that way, influence the response to infection [1,47]. Indeed, the SCFA butyrate was shown to promote the differentiation of bone marrow monocytes from an inflammatory phenotype to a more tolerogenic phenotype [48]. Bone marrow cells also express a variety of PRRs and are susceptible to circulating MAMPs with differential effects dictated by PRR expression and MAMP availability [49]. For instance, activation of the CLR dectin-1 on hematopoietic stem and progenitor cells (HSPCs) leads to induction of trained immunity already described for monocytes and macrophages [50,51]. In contrast, activation of TLR2 on HSPCs rather gives rise to tolerized macrophages with high antigen presentation co-stimulatory capacity [52]. Activation of HSPCs via AhR ligands has been shown to lead to the generation of myeloid-derived suppressor cells capable of immunosuppression [53].

In addition to influencing T cell development and function, gut microbiota-derived signals have also been shown to modulate innate immune defenses by lymphoid stimulation in the spleen, modulation of neutrophil migration and function, induction and activation of macrophages, and stimulation of the maturation of natural killer (NK) cells’ functions [29,39,40,47,54]. More recently, it was shown that specific bacterial species also regulate inflammatory responses via the decrease of plasma corticosterone levels, an anti-inflammatory steroid that is important in the control of the inflammatory response to mucosal injury [55].

Taken together, through the mechanisms described above, it is clear that a dysbiosis in the gut microbiota can lead to a reduced ability to induce a suitable local and systemic immune response, resulting in local inflammatory diseases, but also in diseases at distal sites. One distal site of specific interest is the airways and this specific, direct relationship between these two sites is being referred to as the gut–lung axis [56,57,58]. Indeed, in both animal as well as in human studies, it has been shown that an alteration in the gut microbiota induced by antibiotics can be linked with the development of atopic manifestations, allergic airway disease, and an increased risk to develop asthma [59,60,61,62,63]. Next to impacting the development of allergic airway diseases, it has been shown that the gut microbiota has a crucial role in the protection against bacterial and viral respiratory infections, because the gut microbiota directly steers the innate and adaptive immune response [64,65,66]. Indeed, it has been shown in several human clinical trials that the use of probiotics was associated with a lower incidence and improved health outcome of respiratory infections [67,68,69]. Another mechanism through which events in the gut can impact disease in the lung is through the common mucosal immune system, in which antigen-specific B cells primed in the gut can migrate to a distal effective site via a thoracic duct [57]. It has to be kept in mind though that, in gut–lung microbiota research, as in many other microbiota research fields, it is challenging to determine whether the gut microbiota changes are the cause or the effect of a disease [56]. In addition, longitudinal studies are also needed to gain a better insight into the effect of the gut microbiota on the severity and course of established lung disease.

## 5. Development of the Immune System and the Gut Microbiota in Early Life

Young infants and the elderly are especially vulnerable to infections. One thing these two populations have in common is the fact that, in both these populations, the immune system is not functioning optimally.

The infant’s immune system is not fully functional at birth, meaning both their innate and adaptive immunological responses are greatly suppressed. The in utero environment demands that the fetus’ immune system is actively down-regulated and tolerant to antigens from the mother, in order to avoid immunological reactions that would lead to termination of the pregnancy. However, after birth, the exposure to environmental antigens, many of them derived from intestinal microbiota, requires a rapid change in immune responsiveness to protect the infant from invading pathogens [70]. In the first months of life, protection against many infections is provided by maternal IgG antibody that is being transferred from the mother to the infant; however, when these antibody levels decrease, the infant becomes more vulnerable to infections [71]. Fortunately, innate immune cells, which provide an early first line of defense against invading pathogens, already develop and mature during fetal life; however, this happens at different times, and the function of all components of innate immunity is still weak in newborns compared with later in life. The adaptive immune system also develops rapidly in the first months of life, driven by antigen exposure, which results in the development of immunological memory [70].

The microbiota and the immune system are closely related. Therefore, the increased infection rates observed in infants can also be correlated with changes in the microbiome. An infant may initially be exposed to bacteria in the womb and, after birth, intestinal colonization appears rapidly. The colonization pattern is influenced by, among others, the mode of birth, genetics, whether the infant is being breastfed, geography, and the use of antibiotics [17]. It has been proposed that the first 24 months of life represent a crucial developmental window for the establishment of the microbiome and may even determine the composition of the intestinal microbiota throughout life [72]. As certain bacteria are required for parts of the immune system to develop or mature, these two processes are inextricably linked [73]. Indeed, commensal microorganisms are needed for the immune system to be trained to differentiate between commensal bacteria, which become tolerated antigens, and pathogenic bacteria [74,75]. Defective immunological tolerance promotes exacerbated auto-immune and inflammatory disease, such as allergy [5]. It has been shown that the composition of the intestinal microbiota between atopic and healthy children is different, and reduced bacterial diversity and dysbiosis is associated with the development of atopic diseases [76,77].

## 6. The Aging Immune System and Gut Microbiota

At the other end of the age spectrum, the immune system is also functioning sub-optimally. This biological aging of the immune system, characterized by a progressive decline in both innate and adaptive immunity, is irreversible and is termed “immunosenescence”. Age-associated changes in signaling pathways in dendritic cells (DCs) have been shown to impact their function, leading to an altered cytokine secretion pattern in response to pathogens [78]. In addition, these changes lead to reduced phagocytosis and an impaired ability to present antigens, and negatively impact the ability of DCs to migrate [79]. Similarly, it was shown that circulating monocytes, macrophages, and migratory neutrophils from older people display impaired phagocytosis [80]. In monocytes, DCs, and neutrophils, the expression and function of TLRs decline with age [81,82,83]. In addition, the impaired localization of TLRs can induce a change in cytokine production. One exception is the expression of TLR5 on monocytes from elderly people, which is actually increased compared with TLR5 expression levels found in monocytes from younger individuals and leads to an increased cytokine production in older individuals [84,85]. In addition, intricate alterations occur in T cells with increasing age, including epigenetic and metabolic changes, which affect naïve-, memory-, and effector T cells [86,87]. Moreover, the T cell receptor (TCR) repertoire diminishes and the frequency of senescent or exhausted T cells, which are functionally inactive, increases. The origin of age-associated T cell alterations might lie in adjustments in cytokine production, since cytokines are crucial in mediating T cell responses. It has indeed been shown that T cells from older people mainly show a Th2-like phenotype [88]. There also seems to be an increase in the ratio of Th17 to regulatory T cells, which has been suggested to be associated with a reduced response against infections in the elderly [89]. Next to changes in the T cell compartment, older individuals have a less diverse B cell repertoire, which might contribute to the fact that the elderly are more susceptible to infections.

Immunosenescence is accompanied by a chronic, sterile, low-grade inflammation named inflammaging [90]. There are several activators of the innate immune system that contribute to inflammaging. Such stimuli include persistent viral and bacterial infections, cell breakdown products, and misfolded proteins [91,92,93]. Immunosenescence and inflammaging combined lead to an increased prevalence of infections, cancer, autoimmune and chronic disease, and poor response to vaccination in the elderly [94].

In comparison with gut microbiota research in infants and the effect of the microbiota on the immune system, a smaller number of studies focus on the phylogenetic and functional changes that occur in the gut microbiota during aging. Even though there is a large variability in gut microbiota in older people, the healthy, adult gut microbiota is thought to be rather stable, until the aging process starts to impact the homeostasis of the microbiota [95]. The resulting reduced biodiversity, especially characterized by a reduction in anti-inflammatory SCFA-producing bacteria, as well as compromised stability of the gut microbiota, have often been associated with increased susceptibility to infections [96]. In addition, the chronic low-grade inflammation associated with the changing gut microbiota and immunosenescence favors the growth of pathobionts, a small part of the healthy gut microbiota that, in an inflamed environment, can overtake the growth of symbionts and lead to infection [35].

As the global population is aging rapidly, health in older people is going to be more and more of a concern. Owing to decreased immune function, the elderly are considered to be at increased risk of developing infections, with increased severity and mortality rates compared with younger people. Especially in elderly care facilities, where infections spread easily among residents, preventing infections is crucial [35].

## 7. Infectious Diseases and the Gut Microbiome in the Clinic

Traditionally, the focus of infectious disease specialists has been to recognize and treat individual pathogens. One of the most effective treatment strategies has been the use of antibiotics; however, the rise in antibiotic-resistant pathogens has increased the need for alternative strategies [17,29]. In addition to stimulating the growth of antibiotic-resistant pathogens, antibiotics also disrupt the microbial community structure and function, which allows for potential pathogens to colonize, grow, and persist. As a result of the increased knowledge about the complex balance and interactions between the immune system, the gut microbiota, and pathogens, the field of infectious diseases and clinical microbiology is undergoing a paradigm shift at the moment, and clinicians are now starting to use this scientific information in their clinics [17].

The most eminent example of the relationship between the gut microbiota and infectious diseases is *Clostridium difficile* infection after antibiotic use. During antibiotic treatment, the antibiotic-sensitive bacteria are killed, resulting in reduced signaling of the microbiota and diminished immune responses to *C. difficile* [97]. In addition, *C. difficile* uses the increased amount of nutrients that are available owing to the absence of other bacteria, which leads to an increased colonization rate into sites cleared of bacteria by a range of antibiotics. This strong relationship between the use of antibiotics and *C. difficile* makes this infection an interesting target for microbiome-based therapy [97]. Similarly, multiple enteric viruses, including rotavirus, norovirus, and poliovirus, have been shown to use the bacterial microbiome for immune evasion, supporting entry and replication in the gut, and thus increased infection rates [8,17].

The gut microbiome has the proven potential to influence systemic viral infection, using the systemic immune mechanisms described above. For example, microbiota derived SCFAs have been described to have a protective effect against influenza infection, by modifying T cells’ responses [98]. In addition, a higher abundance of the gut *Lactobacillales* order in HIV patients has been shown to have a negative association with viral load, indicating that the microbiota could, directly or indirectly, modulate the pathology of HIV infections [99].

The microbiome may also influence vaccine responses and drug metabolism, which is currently an area of particular research interest; however, this is likely to be drug- or vaccine-specific [100,101]. In a 2017 systematic review and meta-analysis of randomized controlled trials (RCTs) investigating the effect of prebiotics and probiotics on vaccine immunogenicity and efficacy, thirteen trials using probiotics and six trials using prebiotics were compared [102]. However, the overall result of this meta-analysis should be interpreted with caution, because it combines data on vaccine responses after the use of different prebiotics and probiotic strains. Looking at the effects in individual studies, which sometimes show no effect and sometimes show a positive effect of the intervention, highlights that results are highly intervention dependent [102].

## 8. The Effect of Nutrition on the Gut Microbiome, the Immune System and Infectious Diseases

It is well-understood that nutrition has a large impact on the composition of the gut microbiota and the immune system and can, therefore, play a major role in the development of health and disease [103]. For example, the Western diet has been linked to enhanced inflammatory responses, by inducing epigenetic and transcriptional reprogramming of myeloid progenitors, thereby directly influencing the development of several non-communicable diseases [36]. Increasing our understanding of the relationships between the gut microbiota, host responses, and other microorganisms even further provides opportunities to modulate this triad, for instance, by nutrition, to help sustain intestinal homeostasis and infection resistance. It should be taken into account that different dietary components such as minerals, carbohydrates, vitamins, lipids, and proteins all have specific properties that affect the interaction between the host and a pathogen in a different way, both directly and indirectly through the microbiome. Establishing a mechanistic link between these nutrients provides multiple opportunities to influence health [12]. Dietary intervention should, therefore, be considered a valuable tool to modulate infectious disease risks, prevent the invasion of pathogenic microorganisms, mitigate the severity of infections, and support the treatment of infectious diseases; however, further research in this rapidly emerging field is required [103].

Even though there are multiple nutritional compounds that are known to have an impact on the host microbiome and the immune system, much attention goes out to dietary fibers (DFs), prebiotics, and probiotics.

Prebiotics are nondigestible food ingredients that beneficially affect the host by selectively stimulating the growth and/or activity of one or a limited number of bacteria in the colon [104]. This would mean that not all dietary fibers are prebiotics unless evidence is provided that the fiber is selectively utilized by host organisms conferring a health benefit. Typical prebiotics would be human milk oligosaccharides (HMOs), inulin and fructo-oligosacchardies, and galacto-oligosacharides. Dietary fibers that are not typical prebiotics, but have prebiotic properties are, for instance, beta-glucans, arabinoxylans (AX), pectins, and resistant starches. Prebiotics and specific DFs promote the growth of beneficial bacteria in the gut by acting as a substrate for fermentation and, at the same time, inhibit the growth of pathogens through niche exclusion. The dominant fermentation products are SCFAs, which have a major impact on the immune system, as described above, and can thus inhibit the development of infectious diseases. Besides SCFA, prebiotics and DF may also directly prevent infection of the GI tract through exclusion and antimicrobial activities, as recently reviewed by Asadpoor et al. [105]. In addition, direct interactions of prebiotics and DF with epithelial and immune cells also contribute to preventing infection. DFs such as beta-glucans and AX have been shown to activate the CLR dectin-1, an important receptor involved in the induction of trained immunity, which increases immune responses against secondary infections [51,106,107]. HMOs, AX, and Pectins also interact with TLRs, resulting in increased efficacy of DCs, the induction of tolerogenic DCs via intestinal epithelial cells, and protection of the GI tract from exaggerated TLR signaling, but also support resolution of inflammation following GI infections [108,109,110,111].

Probiotics are live bacteria that, when administered in adequate amounts, provide a health benefit to the host [112]. The rationale for using probiotics is mainly based on their ability to modify the intestinal microbiota, supporting the growth of commensal bacteria over the growth of pathogenic bacteria [113]. Probiotics shape the microbiota by competing with pathogens for nutritional and functional resources and through the production of antimicrobial substances. Many studies have investigated the potential role of probiotics in the prevention and treatment of infectious diseases; however, not all data are in agreement. In a Cochrane systematic review on the efficacy of probiotics in acute diarrhea, the authors concluded that probiotics have clear positive effects by shortening the duration of acute infectious diarrhea and in reducing mean stool frequency [114]. With respect to respiratory tract infections, studies showed that children using probiotics had fewer recurrent respiratory infections in the first year of life, as well as reduced incidences of pneumonia and of severe acute lower respiratory tract infections. However, other studies did not find an effect on the incidence of lower respiratory tract infections [113]. As an example, one study found no difference in the occurrence of otitis media (OM) between the group receiving *Lactobacillus rhamnosus* GG (LGG) and the control group [115]; however, another trial of 72 infants showed that significantly less children that received a combination of LGG and *Bifidobacterium lactis* experienced an episode of OM compared with the control group [116]. Data analyzed from 13 RCTs described in a Cochrane systematic review demonstrated that probiotics significantly reduced the number of episodes of acute upper respiratory tract infections and antibiotic usage [117]. The heterogeneity of data, mainly owing to a variation in strains, doses, study settings, and measured outcomes, limits evidence-based recommendations for the broad use of probiotics to prevent infections.

One of the reasons for conflicting results on the effect of nutritional components, such as pre- and pro-biotics, might be that clinical studies investigating the effect of nutrition are usually designed similar to studies investigating the effect of pharmaceutical compounds. Such a pharma-like approach might not be suitable to identify individual responses to dietary treatment, which might be more multifactorial compared with targeted effects of a pharmaceutical compound. By not reporting individual responses to a nutritional intervention, we might miss out on information that is crucial to better understand the interaction between nutrition, the microbiome, and the host, which would be required to design a personalized nutritional approach [118,119]. Network analysis, systems biology, and machine-based learning techniques that could integrate multiple features based on pre-existing large cohort data sets could provide insights into the effect of specific nutrients on specific health outcomes in an individual. For example, Zeevi et al. has proposed a machine-learning algorithm to predict glycemic responses to real-life meals [120]. However, as the overall health outcome of an individual is dependent on many processes and responses, many large undertakings similar to the endeavor of Zeevi et al. investigating glycemic responses are required to obtain an overall picture of the health status of an individual.

In addition, an individual’s response to a nutritional compound is impacted by the individual’s genetic profile [121]. For instance, single nucleotide polymorphisms (SNPs) in inflammatory genes, such as IL1B, IL6, and TNFA, that lead to a differential inflammatory response could explain part of the observed differences in responsivity to nutritional compounds [122]. There is also increasing evidence that the genetic profile of an individual is of key importance in enabling colonization of the gut with beneficial bacteria, which influences the immune system, overall host health, and infectious diseases.

In addition to using prebiotics and probiotics separately, there are also nutritional concepts in which prebiotics and probiotics are being combined into a synbiotic blend. Taking into account the limitations of clinical studies as described above, synbiotics have shown clinical success and, therefore, hold promise as a treatment option in the future. This became apparently clear in a recent randomized, double-blind, placebo-controlled trial in rural India that showed a significant reduction in sepsis and subsequent death in neonates receiving a seven-day intervention with a synbiotic concept. With sepsis being a major cause of morbidity and mortality in neonates in the developing world, this concept holds great promise to contribute to child health [123].

## 9. Conclusions

As we are learning more about the intricate mechanisms by which the gut microbiota can influence local, innate, and systemic immunity, scientists and clinicians start using this information to develop approaches that target these processes, supporting the ultimate goal of improving prevention and treatment strategies for infectious diseases. Such interventional strategies should take into account the significant variation in both the microbiome and immune responses between individuals, and will thus require a personalized approach. The fact that dietary interventions are able to induce a rapid change in microbiome function and downstream immune responses could be used to develop tailormade nutritional concepts that could influence the development and treatment success of infectious diseases.
